# Predictors of residential stability among homeless young adults: a cohort study

**DOI:** 10.1186/s12889-016-2802-x

**Published:** 2016-02-09

**Authors:** Élise Roy, Marie Robert, Louise Fournier, Émélie Laverdière, Djamal Berbiche, Jean-François Boivin

**Affiliations:** 1 Addiction Research and Study Program, Faculty of Medicine and Health Sciences, Université de Sherbrooke, 150, Place Charles-Le Moyne, room 200, Longueuil, QC J4K 0A8 Canada; 2 Département de Psychoéducation et de Psychologie, Université du Québec en Outaouais, 283, boul. Alexandre-Taché, bureau 3712, C.P. 1250, Succursale Hull, Gatineau, QC J8X 3X7 Canada; 3 École de Santé Publique, Université Montréal, 7101 Avenue du Parc, 3ième étage, Montréal, QC H3N 1X9 Canada; 4 Faculty of Medicine and Health Sciences, Université de Sherbrooke, 150, Place Charles-Le Moyne, room 200, Longueuil, QC J4K 0A8 Canada; 5 Charles-LeMoyne Hospital Research Centre, 150, Place Charles-Le Moyne, room 200, C.P. 11, Longueuil, QC J4K 0A8 Canada; 6 Lady Davis Institute for Medical Research, Jewish General Hospital, 3755, Côte Ste-Catherine, Montréal, QC H3T 1E2 Canada

**Keywords:** Homelessness, Youth, Residential stability, Housing, Cohort

## Abstract

**Background:**

Homelessness episodes have been shown to be associated with serious health outcomes among youth. This study was undertaken to estimate the probability of reaching residential stability over time and to identify predictors of residential stability among homeless young adults aged 18 to 25 years.

**Methods:**

A prospective cohort study was carried out in Montréal, Canada, between April 5^th^ 2006 and January 21^th^ 2009. Interviews conducted every three months included questions on life conditions and social and mental health factors that are known to influence residential trajectories. Residential status was determined, starting on the first day after recruitment; each follow-up day was classified as a homeless day or a housed day. A period of 90 days was used to define residential stability; therefore the main study outcome was the occurrence of the first consecutive 90 housed days during the follow-up period. Kaplan-Meier and Cox proportional-hazards regression analyses were conducted.

**Results:**

Of the 359 participants, 284 reached 90 days of residential stability over the study period, representing an annual probability of 80.5 %. In multivariate analysis, youth who had a high school degree, had a formal sector activity, and those who had sought psychological help were more likely to reach residential stability. Being a man, injecting substances, and having an informal sector activity were associated with a decreased probability to reach residential stability.

**Conclusion:**

Exposure to factors related to opportunities that promote social integration increases the chance of reaching residential stability. On the other hand, factors related to high level of street entrenchment seem to interfere with stabilization. Maximum efforts should be made to prevent chronic homelessness among youth, targeting not only individual impairments but also hinging on services adapted to foster social connections among the youth.

## Background

Homelessness has deleterious effects on the development and health of youth. Precarious living conditions, hostile social environments, poor access to services, even just the struggle of day-to-day street survival are some of the main factors that significantly contribute to those effects. As a result, there is an increased risk of death: reported standardized mortality ratios show that mortality rates for homeless youth are 2.7 to 37.3 times higher than for other young people [1].

Studies have shown that street youth experience major residential transitions over relatively short time periods, alternating between lack of any housing, extremely precarious housing, and stable and autonomous housing [2–4]. Homelessness is a dynamic phenomenon; thus longitudinal designs are of paramount importance when studying the course of housing and its determinants [5]. Yet, only a few longitudinal studies have allowed quantitative examination of residential trajectories of homeless youth either adolescents or young adults [6–13]. Of these, five included adolescents [7–11] and two [7, 10] focused specifically on newly homeless youth. Although undoubtedly valuable, those studies do not specifically consider the situation of young adults. Indeed the course of homelessness among young adults remains poorly documented despite the many characteristics distinguishing them from adolescents and from older street-involved populations. It is well acknowledged that the young adult or late adolescent stage is crucial to a normal outcome in adult functioning [14]. During this stage, many developmental tasks need to be completed to successfully transition to adulthood. Social factors clearly influence the probability of a successful transition in terms of self‐sufficiency. One of these is certainly homelessness where young people living in unstable housing are more likely to experience living conditions that can hinder adult development [15]. Increased understanding of residential trajectories of homeless young adults will help formulate better public health interventions related to housing. We report below the results of a prospective cohort study which objectives were to estimate the probability of reaching residential stability over time and to identify predictors of residential stability among homeless young adults.

## Methods

Between April 2006 and May 2007, study interviewers recruited participants through regular visits to all major street youth agencies in Montréal, Canada. As in our previous studies [1], youth were considered street-involved if they had used the services of Montréal street youth agencies at least three times in the previous year or had been without a place to sleep more than once during the same period. Only street youth who had experienced at least one 24-h episode of homelessness in the previous 30 days were eligible for this study. Such episode was defined as having spent at least one night in a place unfit for human habitation or having been housed temporarily in an emergency shelter or with friends or acquaintances. Other eligibility criteria included being 18 to 25 years of age, speaking French or English, being able to provide informed consent and to complete an interviewer-administered questionnaire, and planning to stay in the Montréal area for the following year.

The initial interview included signing a consent form and providing contact information. Six follow-up interviews took place every three months until January 2009. Detailed contact information was updated at each interview and thorough follow-up procedures were used. Participants received financial compensation (CAD $30) for each interview. This study was conducted with the approval of the *Comité d’éthique de la recherche en santé chez l’humain du Centre Hospitalier Universitaire de Sherbrooke et de l’Université de Sherbrooke* and conformed to the principles embodied in the Declaration of Helsinki.

### Measurements

All questionnaires were administered by interviewers trained specifically for this study. A questionnaire based on the “Life History Calendar” technique [16] and the residential follow-back calendar [17, 18] was used to document the main outcome of this study. Residential status was assessed day-by-day for the whole period since the previous interview (or during the 3 months prior to the intake interview). To help participants remember their housing status by situating them in time, the interview started with questions about recent significant life events. Participants were asked if they had experienced any of 33 positive or negative life events. This information was then noted on a calendar and used to guide the interview documenting the participant’s sleeping arrangements and locations on a daily basis for the whole time period. Based on this information, residential status was determined for each follow-up day, starting on the first day after recruitment (referred to as Day 1). Each follow-up day was classified as a homeless day or a housed day, the latter corresponding to the following situations: the youth resided (1) in his or her own home; (2) in his or her partner’s home or with his or her parents or with relatives, friends, acquaintances, or families of friends without the reason being a need for temporary assistance; (3) in housing resources (excluding emergency or short-term shelters); (4) in a campground, hotel or motel (not as an emergency measure); or (5) in a place where a person works and lives (e.g., farm, fairground). Youth spending a day in transitional facilities such as a police station, jail, prison, correctional halfway house, hospital, detoxification or rehabilitation center, or other similar resources were considered as housed on these days if these stays had been preceded by housed days; otherwise, these days were considered as homeless days. Further details about our research methodology and study instruments have been previously described [19].

As for exposure variables, we focused on life conditions and social and mental health factors that are known to influence residential trajectories among homeless people [3–5, 20]. Each interview included questions about socio-demographic variables, previous homelessness experience, use of formal resources to get off the street, involvement in formal and informal sectors of activity, experience with the justice system, personal social network, psychological distress, suicidal thoughts and attempts, and markers of intensive drug use including experience of drug overdose and injection drug use. Substance use disorders and other mental health diagnoses were measured only at baseline.

Formal sector activity was defined as having at least one source of income among the following during the last 3 months: full-time job, part-time job, occasional jobs, having a welfare cheque or unemployment cheque, and loans and grants from government sources. Informal sector activities included sources of income from family or friends, prostitution, procuring *(pimping)*, sale of personal property (e.g., pawn shop), sale of drugs, artistic performances on the street or in the subway, begging, squeegeeing, and theft, fraud or concealment.

Formal resources to get off the street included community-based or institutional services providing help finding a job, obtaining financial or legal assistance, and finding housing during the last 3 months. Experience with the justice system was defined as involvement in at least one of the following situations during the last 3 months: police arrest, awaiting trial or sentencing, having sentences with probation, being on conditional release, being in detention or owing money after getting a ticket. Having a social network at risk was defined as currently having regular interactions with persons (i.e., boyfriend, girlfriend or anyone else excluding formal resource providers) who have problems with alcohol or drugs, having sex in exchange for money or drugs, injecting drugs or being homeless. The presence (in the last three months) of support from family, friends or boyfriend/girlfriend in the form of emotional support, instrumental support, advice or protection was also examined at every visit.

Psychological distress was assessed at each visit by the K10 scale developed and validated by Kessler and coll [21]. It consists of 10 questions on non-specific psychological distress symptoms a person may have experienced in the most recent four-week period. The final score ranges between 10 and 50 and a score equal or greater than 30 was considered as severe psychological distress. Questions about diagnoses of major depression, bipolar disorders, anorexia/bulimia, and schizophrenia were taken from the World Mental Health Composite International Diagnostic Interview (CIDI) version 2.1. [22]. Anxiety and alcohol or drug-related disorders were assessed using the simplified version (CIDIS) developed by Kovess and colleagues [23]. The CIDI and the CIDIS are well-validated tools that can be administered by lay interviewers and produce psychiatric diagnoses according to the fourth version of the Diagnostic and Statistical Manual of Mental Health Disorders published by the American Psychiatric Association [24]. Having at least two mental health disorders was defined as demonstrating two or more of the following diagnoses: major depression, bipolar disorder, anorexia/bulimia, schizophrenia or anxiety.

## Analyses

Frequency distributions were calculated to characterize the sample with respect to baseline characteristics. A period of 90 days was used to define residential stability, and therefore, only subjects who completed at least 90 days of follow-up were included in the analyses. A 90-day period was considered sufficiently long to represent significant stability; it was also comparable to the minimum timeframe used in the most recent literature [11]. More specifically, 90 days of residential stability was defined as the occurrence of the first consecutive 90 housed days during the follow-up period. The occurrence of this event (i.e., 90 days of residential stability) was defined as happening on *day i* if the 90 days following *day i* represented 90 consecutive housed days. Using the Kaplan-Meier method, we estimated the cumulative probability of youth returning to a housed status for 90 consecutive days during follow-up. The probability of reaching a first episode of 90-day residential stability over one year was also computed using the following formula: 1-e^-mean per-person incidence rate*length of observation^ [25]. Predictors of 90-day residential stability were assessed using Cox proportional-hazards regression. The measures of these predictors were obtained from questionnaires on *Day i* or previously.

Univariate analyses were conducted and factors with *p*-values less than 0.20 were retained for multivariate analyses. Following the purposeful selection procedure, significant variables at a level alpha of 0.05 and those with a confounding effect were retained in the final model. A variable was considered as a confounder if its removal from the model changed a significant coefficient by more than 20 %. SAS 9.3 software was used to perform the analyses.

## Results

**Fig. 1:**
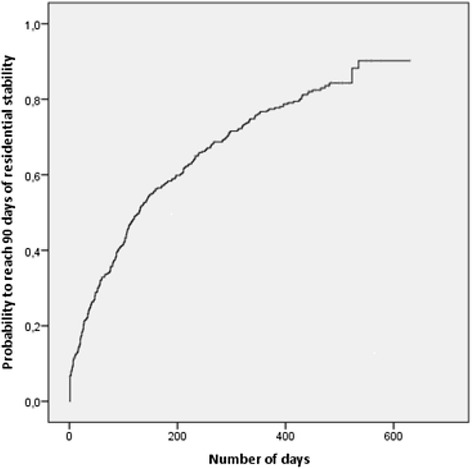
Cumulative probability to reach a housed status for 90 consecutive days during follow-up

Of the 419 youth recruited, 359 (85.7 %) completed at least 90 days of follow-up, corresponding to 64,479 days of follow-up at risk (mean 180 days; range: 1–630, median 114 days). Two hundred and eighty-four participants (79.1 %) reached 90 days of residential stability over the study period, representing an annual probability of 80.5 %. The median survival time was 129 days and the corresponding interquartile range was 39 to 343 (Fig. 1).

Most participants were men (79 %) and median age at study entry was 22 years (Table 1). At baseline, almost three-quarters of the participants reported having been homeless during their lives (not necessarily continuously) for more than a year (median: 2.00, IQR : 0.75–4.00). In all, 143 subjects presented at least one mental health problem in the last year (40 %).

**Table 1:** Characteristics of study participants at baseline (*n* = 359)

	Number of subjects at baseline (%)
Baseline variables	
Male	284 (79.1)
Age at first homelessness episode (≥16 years old)	222 (61.8)
Age (≥22 years)	182 (50.7)
Lifetime cumulative homelessness (≥1 year)	259 (72.1)
Alcohol abuse or dependence (last 12 months)	119 (33.2)
Drug abuse or dependence (last 12 months)	233 (64.9)
Mental health problem (excluding abuse or dependence to alcohol or drugs; last 12 months)	143 (39.9)
≥2 mental health problems (last 12 months)	73 (20.3)
Time-dependent variables	
High school or more	85 (23.7)
Formal sector activity (last 3 months)	120 (33.4)
Informal sector activity (last 3 months)	315 (87.7)
Experience with the justice system (last 3 months)	277 (77.2)
High psychological distress (last month)	87 (24.2)
Social network at risk (currently)	308 (86.0)
Formal resources to get off the street (last 3 months)	184 (51.3)
Formal resource providing help to find housing (last 3 months)	72 (20.1)
Seeking psychological help (last 3 months)	135 (37.6)
Program or therapy for alcohol or drugs (last 3 months)	60 (16.7)
Active support from family, friends or boyfriend/girlfriend (last 3 months)	315 (91.8)
Active support from a formal individual (last 3 months)	118 (34.4)
Suicidal ideation (last 3 months)	55 (15.3)
Attempted suicide or intentional overdose (last 3 months)	20 (5.6)
Accidental overdose (last 3 months)	26 (7.2)
Injection (last 3 months)	78 (21.7)

Results of univariate and multivariate Cox proportional-hazards regression analyses are shown in Table 2. Youth who had a high school degree, had a formal sector activity, and those who had sought psychological help were more likely to reach residential stability compared to those who did not. On the other hand, being a man, injecting substances, and having an informal sector activity were associated with a decreased probability to reach residential stability in multivariate analysis. Of note, participants excluded from the analyses (*n* = 60) were similar to those included with respect to all predictors identified except for injection drug use where 22 % of participants reported drug injection compared to 10 % of non-participants (*p* = 0.036).

**Table 2:** Crude and multivariate Cox proportional-hazards regression of predictors of 90-days residential stability (*n* = 359)

	Crude HR (95 % CI)^b^	AHR^a^ (95 % CI)
Baseline variables		
Male	0.763 (0.579 - 1.004)	0.726 (0.545–0.969)
Age at first homelessness episode (≥16 years old)	1.247 (0.979 - 1.587)	-
Lifetime cumulative homelessness (≥1 year)	0.810 (0.627 - 1.047)	-
Drug abuse or dependence (last 12 months)	0.761 (0.598 - 0.968)	-
≥2 mental health problems (last 12 months)	1.319 (0.997 - 1.744)	-
Time-dependent variables		
High school or more	1.501 (1.149 - 1.962)	1.470 (1.122–1.926)
Formal sector activity (last 3 months)	1.659 (1.308 - 2.104)	1.507 (1.181–1.924)
Informal sector activity (last 3 months)	0.632 (0.461 - 0.866)	0.674 (0.488–0.931)
Consultation for emotional or nervous problems (last 3 months)	1.377 (1.085 - 1.747)	1.359 (1.069–1.727)
Injection (last 3 months)	0.665 (0.494 - 0.894)	0.692 (0.507–0.944)

^a^ *AHR* Adjusted Hazard Ratio

^b^Only the variables with *p*-value ≤0.20 were included in the multivariate analysis

## Discussion

Our study is the first to prospectively examine the proximal predictors of residential stability in a cohort of homeless young adults. It is also the first to define the outcome using number of consecutive days of housing in places fit for human habitation during the study period. Only a few prospective studies have looked at residential stability among homeless youth and none have determined housing status on a day-to-day basis [7–10, 13]. Various measures of housing and residential trajectories have been reported, all based on questions about current housing status at time of interview or recall of various living situations since last interview. Given these significant methodological differences, it is difficult to compare our data to existing literature. Furthermore, the notion of stability considered in our study did not take into account qualitative aspects of housing such as satisfaction and security [26]. However, our study results, as those of similar studies, point to the same conclusion: stability defined on the basis of lasting episodes of housing is a reachable goal for many homeless youth, at least for a three-month period.

An important finding is that the annual probability of reaching at least one episode of 90 consecutive housed days during follow-up was high (80.5 %). Although this is very good news, our figures show that for half of the study participants, the first 90-day period of stability began after quite a long period, that is, 129 days or 4.3 months after recruitment. In addition, a majority of participants already had a significant lifetime homelessness experience at study entry. The data suggest that in our sample, homelessness has not been a short term event but rather a relatively long-lasting issue. It is interesting to note that among factors leading to stability, a number of them concern access to resources that Slescnik et al. call the social system (employment, school and medical care) [9]. Youth who had sought psychological assistance, who had earned a high school diploma and who were working during follow-up were 40 to 50 % more likely to achieve stability than the other youth. These results are consistent with those of studies in the United States looking at samples that are comparable, in terms of age. In their longitudinal study of homeless youth in California, Tevendale et al. found that less involvement in informal sector activities predicted membership in the “consistently sheltered” versus “inconsistently sheltered” group during follow-up [11]. Moreover being able to go back home was more important than degree of individual impairment such as substance use and mental health problems. In their Ohio study, Slesnick et al. concluded that youth who had more social connections at the beginning of the study were more likely to see their number of homeless days decrease during follow-up [9]. In our study, while background characteristics such as lifetime duration of homelessness or diagnoses of substance use or mental health disorders were not associated with the outcome, exposure to factors related to opportunities that promote social integration during follow-up was key. This demonstrates the importance of providing services adapted to the needs of young people so they can build up confidence and develop skills to engage in society, which in turn encourage them to get off the streets.

Our results show that drug injection reduces by 30 % the chances of having at least one episode of residential stability during the follow-up period. The link between problematic substance use and homelessness is complex. The literature suggests there is a reciprocal relationship, where worsening of housing conditions can lead to increased consumption that can then jeopardize the capacity of achieving housing stability [19, 27]. Therefore, it is difficult to determine the direction of the causal link, if any. More specific studies on drug injection among street youth have shown that homelessness episodes increase the risk of initiation into injection drug use in this population [28, 29]. The question as to whether drug injection perpetuates homelessness remains poorly documented. It is plausible, however, considering that injection is a very intense way of consuming drugs. Similarly, intense drug consumption was found to be negatively associated with transitioning out of homelessness among street-involved youth in Vancouver [13]. Indeed, intensive use is often associated with a lifestyle linked to high level of street entrenchment [30, 31] which, in turns, interferes with reintegration into mainstream society. Likewise, being involved in informal sector activities also decreased the risk of reaching residential stability among our study participants.

Finally, being female predicted a greater likelihood of attaining a first episode of stability, although the association was marginally significant. Similarly Tevendale et al.’s study showed that females were more likely than males to follow “consistently” and “short term inconsistently sheltered” trajectories as opposed to a “late term consistently sheltered” trajectory. However this association no longer applied in the multivariate analysis. The relatively small number of women typically observed in cohorts of homeless makes it difficult to estimate the probability of reaching residential stability according to gender. Yet the literature suggests that the experience of women and men is different which could influence the probability of stabilizing. For example, while it is generally acknowledged that young street women are more victimized than males, they also make more use of their social networks than young men to cope with homelessness [32]. In the first case, this could compromise the stabilization process while in the second case, this could be the reverse.

Our study presents a number of strengths and limitations. The instrument we used to assess the study outcome was adapted from the Residential Follow-Back Calendar designed by the New Hampshire Dartmouth Research Center [18]. Tsemberis et al. [17] assessed the psychometric properties of the Calendar and demonstrated its high test-retest reliability, sensitivity to change and concurrent validity. In terms of limitations, our data collection method was based on self-reports, which may have introduced the possibility of both recall and social desirability biases. The impact of such biases was however limited by our use of the Life History Calendar technique, the short time spans between interviews (3 months), and the interviewers’ open and non-judgmental attitudes. Secondly, our decision to focus on life conditions and social and mental health factors that could predict residential stability in the course of homelessness might have prevented us to identify important background factors such as childhood-related factors. Thirdly, our results may not be generalizable to all street youth. The majority of participants already had a relatively long history of homelessness at recruitment. Despite their young age, many of them had been involved in the street economy, arrested or convicted, and reported high risk networks, suggesting an already significant level of street-entrenchment at baseline. Finally, keeping up with homeless populations can be particularly challenging. In our study, 87 % of subjects recruited at baseline were re-interviewed at three months, 72 % at 12 months, and 45 % at 18 months. These figures are comparable to that of Tevendale et al., who reported a two-year follow-up percentage of 42 %. Losses to follow-up may have affected our estimates of residential stability, especially after 12 months of follow-up. On one hand, it is plausible that subjects who stabilized for long periods dropped out of our study more frequently than less stable youth, leading to deflated housing stability estimates. On the other hand, it is conceivable that subjects who did not stabilize were more likely to be lost to follow-up due to chaotic lifestyles that jeopardized their continued participation. It is less clear that the correlate analysis could be biased. To affect the magnitude or direction of risk ratio estimates, drop-out rates would have to differ according to presence of both a correlate and housing stability [33].

Some comments are warranted concerning the fact that having received formal support to find housing was not a significant predictor of the study outcome. It is well acknowledged that securing housing for homeless people provides the stability often needed to access education, employment and all other assets necessary to develop an independent life. We do not believe these results should be interpreted as conflicting with the literature. In fact, at time of study, supportive youth-focused housing resources were very limited in Montréal. This could have affected our power to detect an association.

## Conclusion

The results of this study are encouraging in that most homeless youth achieve residential stability even after relatively long stays in the street. Our findings suggest that efforts to prevent chronic homelessness among youth should not only target individual impairments but also build on services that foster social connections among the youth.
